# Comparison between LigaSure™ and Harmonic® in Laparoscopic Sleeve Gastrectomy: A Single-Center Experience on 422 Patients

**DOI:** 10.1155/2019/3402137

**Published:** 2019-01-03

**Authors:** N. Velotti, M. Manigrasso, K. Di Lauro, A. Vitiello, G. Berardi, D. Manzolillo, P. Anoldo, A. Bocchetti, F. Milone, M. Milone, G. D. De Palma, M. Musella

**Affiliations:** Department of Advanced Biomedical Sciences, “Federico II” University, Via Pansini 5, 80131 Naples, Italy

## Abstract

**Background:**

New laparoscopic devices, such as electrothermal bipolar-activated devices (LigaSure™ (LS)) or ultrasonic systems (Harmonic® scalpel (HS)), have been applied recently to bariatric surgery allowing to reduce blood loss and surgical risks. The aim of this study was to retrospectively compare intraoperative performance of HS and LS, postoperative results, and clinical outcomes in a large cohort of patients undergoing LSG.

**Methods:**

Data from 422 morbidly obese patients undergoing LSG in our Bariatric Unit at the Advanced Biomedical Sciences Department of the “Federico II” University of Naples (Italy) between January 2009 and December 2017 were retrospectively analyzed. Subjects were divided into two groups (HS and LS), and operative time, intraoperative complications, and postoperative (within 30 days from surgery) complications were compared. Bleeding from the omentum or from the staple line, use of hemostatic clips, and absorbable hemostat were recorded as intraoperative complications; hemorrhages, abscess formation, gastric leaks, fever, and mortality were considered as postoperative complications.

**Results:**

Statistical analysis showed no difference in terms of baseline demographics between the two cohorts. Operative time (48 ± 9 vs 49 ± 6 min, *p*=0.646) and the rates of intraoperative and postoperative complications did not significantly differ between groups.

**Conclusion:**

Harmonic® and LigaSure™ are both useful tools in bariatric surgery, and these two advanced power devices are user-friendly and can facilitate surgeon work; from this point of view, the choice of the energy device should be based on the preference of the surgeon and on the hospital costs policy and availability.

## 1. Introduction

Laparoscopic sleeve gastrectomy (LSG) was conceived as the first surgical step for high-risk patients undergoing laparoscopic Roux-en-Y gastric bypass (LRYGB) or biliopancreatic diversion with duodenal switch (BPD-DS) [1, 2]. This bariatric procedure has gained popularity because of its relative simplicity and great results shown over the years, both on weight loss- and on obesity-related comorbidities [3–5]. Although LSG is commonly considered a safe and effective procedure, some complications may occur during and after surgery such as bleeding, staple line leaks, and micronutrient deficiencies [6–11].

New laparoscopic devices, such as electrothermal bipolar-activated devices (LigaSure™ (LS)) or ultrasonic systems (Harmonic® scalpel (HS)), have been applied recently to bariatric surgery allowing to reduce blood loss and surgical risks. These instruments, used to achieve an adequate hemostasis and an easier tissue dissection, have become coresponsible of the short learning curve and of technical simplicity of LSG.

LigaSure™ (Valleylab, Boulder, CO, USA) is an electrothermal device which is used to seal vessels up to 7 mm in diameter. Its form of energy denatures collagen and elastin of vessels and connective tissue determining vessel fusion. It is important that a feedback-controlled response system automatically discontinues energy delivery when the seal cycle is complete. Harmonic Ace® (Ethicon Endo-Surgery, Inc.), instead, uses ultrasonic vibration determining effects of cutting, coaptation, coagulation, and cavitation of vessels tissues. It is documented that it produces minimal lateral thermal spread when dissecting near vital structures [12, 13].

The aim of this study was to retrospectively compare intraoperative performance, postoperative results, and clinical outcomes of HS and LS in a large cohort of patients undergoing LSG.

## 2. Methods

Data from 422 morbidly obese patients undergoing LSG in our Bariatric Unit at the Advanced Biomedical Sciences Department of the “Federico II” University of Naples (Italy) between January 2009 and December 2017 were retrospectively analyzed. Subjects were divided into two groups (HS and LS); baseline demographics, such as gender, age, height, weight, comorbidities, and previous operations were recorded. Operative time and intraoperative and postoperative (within 30 days from surgery) complications were compared. Bleeding from the omentum or from the staple line and use of hemostatic clips and absorbable hemostat were recorded as intraoperative complications; hemorrhages, abscess formation, gastric leaks, fever, and mortality were considered as postoperative complications. Patients were men and women aged between 18 and 65 years with a body mass index (BMI) ranging from 35 to 55 kg/m^2^. Criteria of exclusion from the study were previous supramesocolic surgery, ASA (American Society of Anesthesiology) score 4, treated or untreated malignancies at any stage, and conversion to open surgery.

The study was approved by our institutional review board, and informed consent was obtained from all subjects before enrollment. All investigations complied with the principles of the Declaration of Helsinki (64th WMA General Assembly, Fortaleza, Brazil, October 2013).

### 2.1. Preoperative Care

All patients underwent a preoperative esophagogastroscopy (EGDS) to rule out gastric lesions, and a pulmonary thromboembolism (PE) prophylaxis was administered according to SICOB (Italian Society of Bariatric Surgery) guidelines [14]. Perioperative antiplatelet drugs administration was managed according to validated criteria [15]. One dose of 2 g ceftriaxone was administered intravenously 10–15 min before the operation for infection prophylaxis. In all cases, surgery started with a laparoscopic approach. All patients had the same protocols for anesthesia and postoperative management.

### 2.2. Statistical Analysis

Statistical analysis was performed with the Statistical Package for Social Sciences (Version 20.0 for Windows; SPSS Inc, Chicago, Ill, USA). The Mann–Whitney *U* test was used for comparison between two categories of a categorical variable, and Pearson's chi-square was used in order to evaluate any association between pairs of categorical variables. A *p* value of <0.05 was considered statistically significant.

### 2.3. Surgical Technique

All operations were performed by the same two experienced bariatric surgeons (Ma. Mu, Ma. Mil).

The choice of the device (LS or HA) used during surgery was based on the availability of our clinic; both surgeons used both devices from the beginning of the learning curve.

Following the preparation of the greater curvature, a gastric sleeve was tailored using a 60 mm linear stapler (Echelon flex 60®, Ethicon Endo-Surgery, Johnson & Johnson©, Somerville NJ, USA) and a 38F bougie. A total of five to seven cartridges were used. Between the closure of the stapler and its firing, a 20 sec interval was observed in any case [16]. A methylene blue test with 80–100 mL of saline solution was routinely performed to evaluate possible leaks. No oversewing of the staple line was performed to prevent bleeding or staple line leaks, but human fibrin sealant (Tisseel™, Baxter® Deerfield, IL, USA) was sprayed along the suture line [17, 18]. The excided stomach was extracted as previously described [19]. In all patients, a nasogastric tube and a drainage tube were positioned at the end of the procedure. The nasogastric tube was removed on postoperative day (POD) 1, and a liquid diet was started on POD 3 and was allowed for 10–15 days under strict nutritionist surveillance [20]. An abdominal CT scan was scheduled if clinical symptoms (fever, tachycardia, leukocytosis, and pain) were present [21]. Patients were routinely discharged on POD 5.

## 3. Results

During the study period, 422 patients underwent laparoscopic sleeve gastrectomy for morbid obesity in our institution; 108 (25.6%) were men and 314 (74.4%) were women; two hundred twenty-five of them were operated using LS, and the other 197 were operated using HS.

Statistical analysis showed no difference in terms of age, BMI, comorbidities (diabetes and hypertension), and length of stay between the two cohorts; no differences were found in the number of cartridges used during surgery (Table 1).

Operative time at the beginning of our learning curve (first 25 cases operated with LS vs first 25 cases operated with HA) did not significantly differ between groups (159 ± 7 vs 161 ± 9 min, *p*=0.384); the same results were observed when considering operative time in last 25 cases operated with LS vs last 25 cases operated with HA (48 ± 9 vs 49 ± 6 min, *p*=0.646).

**Figure 1:**
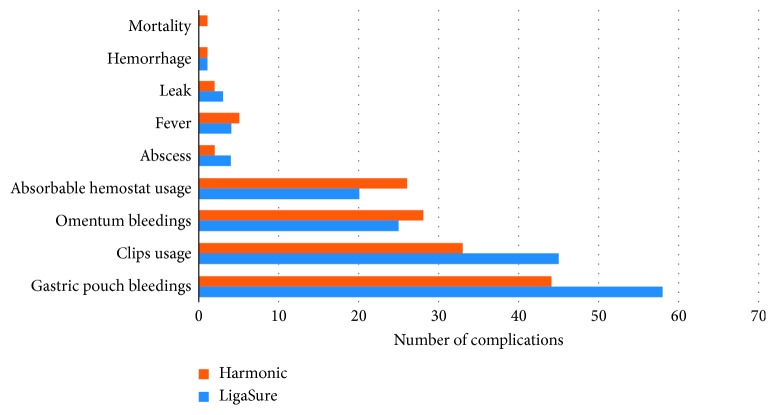
Intraoperative and postoperative complications occurred with Harmonic® and LigaSure™ in the patients' cohort (within 30 days).

**Figure 2:**
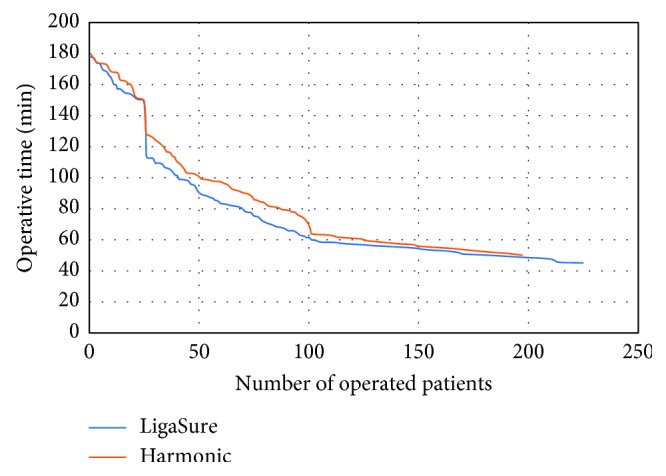
Differences in operative time between Harmonic® and LigaSure™.

Finally, the rates of intraoperative and postoperative complications and the number of surgical revisions did not significantly differ between groups (Table 2) (Figures 1 and 2).

## 4. Discussion

LSG is usually considered as a restrictive procedure, and even though some studies have speculated about the metabolic effects on gut hormones release, this issue remains controversial [22]. LSG may entail higher operative and perioperative risks in comparison with other purely restrictive procedures [23]; nevertheless in skilled hands, its efficacy remains undisputed, especially in the long term, presenting a very low rate of major complications. [24].

Nowadays, different methods are available to control bleeding during laparoscopic procedures: energy devices, electrocautery, clips, vascular staplers, and intracorporeal sutures. Electrocautery is cost-effective and easy to access, but it offers less hemostatic effect when compared to bipolar or energy‐based vessel-sealing devices (VSDs) and causes more lateral thermal damage in the peripheral tissues [25].

Vessel sealing devices differ in design and in the type of energy used; the first generation of ultrasonic VSDs was able to seal vessels up to 3 mm but currently, technological innovation allowed these instruments to close structures up to 7 mm [26]. Okhunov et al. [27] found there are no bursting pressure failures for the HA and LS up to 9 mm vessels.

In order to choose an adequate device to minimize intraoperative and perioperative surgical complications, we compared two different power devices, which were both widely used in various kinds of surgeries.

Many studies in the literature have compared the effectiveness of these two devices in endocrine, colorectal, and gynaecological surgery, but evidences from randomised studies, particularly in laparoscopic approach, are very limited [28–32].

Previous studies on the topic have shown comparable advantages of the use of LA and HA. Campagnacci et al. [33] found no difference in the duration for colorectal surgery between the two devices, but there was less bleeding with LS. No statistical difference was also detected by Yavuz et al. [34] in a randomised trial with 24 cases of laparoscopic appendicectomy. Rimonda et al. [35] analyzed results from 140 patients (31 right hemicolectomies, 69 left hemicolectomies, and 40 anterior resections of rectum): they concluded that LigaSure and Harmonic are both useful and safe instruments for laparoscopic colorectal surgery with no significant difference in terms of intraoperative/postoperative morbidity and operative time.

About bariatric surgery, Tamis et al. [36], in an RCT on 94 patients who underwent LSG using LS or HA, found no significant differences in operative time and complications and concluded that the choice between these two shears lies with the surgeon's preference.

To the best of our knowledge, this study involves the largest series of patients undergoing LSG in which differences between Harmonic® and LigaSure™ use is analyzed, and the results we obtained failed to show a clear advantage in favor of one of the two devices.

The difference in operative time observed in favor of LigaSure™, although not significant, seems to depend principally on surgical dissecting difficulties observed in some patients treated with HA, but these adverse events did not lead to any clinical consequence in the postoperative course. Moreover, the risk of intraoperative bleeding, the use of hemostatic clips and absorbable hemostat, and the postoperative complication rate were similar in the two groups. By this point of view, all bleedings were managed conservatively except for 2 cases in the LS group and 1 case in the HA group which needed a surgical revision.

The only case of death following surgery has been determined by an acute leak on POD1 which caused a rapidly evolving septic shock.

The main limitation of our study is represented by its retrospective design; furthermore, at the beginning of our experience with LSG, operative time was not reported following the video recording which is currently available. This may have generated some inaccuracy in the evaluation of operative time. In contrast, we included a large number of cases, all of which underwent LSG by the same two surgeons at a single institution, minimizing the possible bias induced by surgeons' expertise.

## 5. Conclusions

Our findings suggest that Harmonic® and LigaSure™ are both useful in bariatric surgery, and these two advanced power devices are user-friendly and can reduce surgeon work load; from this point of view, the choice of the energy device should be based on the preference of the surgeon and on the hospital cost policy.

## Figures and Tables

**Table 1:** Characteristics of study population.

	LigaSure™ (*n*=225)	Harmonic Ace® (*n*=197)	*p* value
Female (*n*)	175	139	0.090
Age (years)	42.6 ± 10.73	41.2 ± 8.26	0.131
BMI (kg/m^2^)	47.2 ± 6.11	47.7 ± 5.23	0.365
Diabetes (*n*)	81	77	0.513
Hypertension (*n*)	67	75	0.072
Length of stay (days)	4.8 ± 1.3	5.1 ± 2.1	0.074
Cartridge used (*n*)	6.51 ± 0.79	6.56 ± 0.77	0.512

**Table 2:** Intraoperative and perioperative complications of the patients who underwent laparoscopic sleeve gastrectomy with LigaSure™ and Harmonic Ace®.

	LigaSure™	Harmonic Ace®	*p* value
*Intraoperative complications (n)*			
Omentum bleedings	25	28	0.337
Staple line bleedings	58	44	0.409
Clips	45	33	0.391
Absorbable hemostat	20	26	0.156
*Postoperative complications (n)*			
Hemorrhage	1	1	0.924
Abscess	4	2	0.509
Leak	3	2	0.763
Fever	4	5	0.589
Mortality	0	1	0.284
*Complications needing revision (n)*	2	1	0.584

## Data Availability

The data used to support the findings of this study are available from the corresponding author upon request.
